# Congenital Anomalies among Offspring of Women Living in Low Socioeconomic Status Neighborhoods or Hispanic/Latino Enclaves, Texas, 1999-2018

**DOI:** 10.23889/ijpds.v9i5.2742

**Published:** 2024-09-18

**Authors:** Jeremy Schraw, Elwin Jaime, Rutu Rathod, Charles Shumate, Amy Hughes, Sandi Pruitt, Gary Shaw, Philip Lupo

**Affiliations:** 1 Baylor College of Medicine; 2 University of Texas Southwestern Medical Center; 3 Texas Department of State Health Services; 4 Stanford University

## Objectives and Approach

Neighborhood influences pregnancy outcomes through effects on maternal health. We evaluated prevalence of >140 congenital anomalies monitored by the population-based Texas Birth Defects Registry among residents of Hispanic/Latino enclaves (census tracts with high proportions of Hispanic/Latino residents, immigrants, and Spanish-speaking households) and tracts with low socioeconomic status (low nSES). We included all cases regardless of pregnancy outcome (1999-2018) and a reference population of all livebirths, identified by linkage to Texas vital records. We calculated Yost socioeconomic index and Hispanic/Latino enclave index scores using linked U.S. Census Bureau data. We used Poisson regression to estimate the prevalence ratio (PR) and 95% confidence interval of anomalies among residents of: low nSES/enclave (34.5%); low nSES/non-enclave (25.6%); high nSES/enclave (4.6%); and high nSES/non-enclave (referent; 35.3%) tracts. We adjusted for maternal age, education, race/ethnicity, parity, and pre-pregnancy body mass index.

## Results

Low nSES was associated with microcephaly, pulmonary artery anomalies, atrial septal defect, and Down syndrome (PRs 1.1-1.6), and inversely associated with hypospadias and other male genital anomalies (PRs 0.8-0.9). Offspring of women in low nSES enclaves were at increased risk of several congenital heart defects relative to those in other neighborhoods. Conversely, prevalence of hypospadias was lowest among offspring of women in low nSES enclaves.

## Conclusions

Linkage between a population-based registry, vital records, and Census Bureau data revealed novel associations between neighborhood characteristics and congenital anomalies.

## Implications

Further research is warranted to understand mechanisms linking neighborhood factors to congenital anomalies.

